# Interferon-β deficiency at asthma exacerbation promotes MLKL mediated necroptosis

**DOI:** 10.1038/s41598-018-22557-6

**Published:** 2018-03-09

**Authors:** Samuel C. Cerps, Mandy Menzel, Irma Mahmutovic Persson, Leif Bjermer, Hamid Akbarshahi, Lena Uller

**Affiliations:** 1 Unit of Respiratory Immunopharmacology, Department of Experimental Medicine, Lund University, Lund, Sweden; 2 Respiratory Medicine and Allergology, Lund University, Lund, Sweden

## Abstract

Defective production of antiviral interferon (IFN)-β is thought to contribute to rhinovirus-induced asthma exacerbations. These exacerbations are associated with elevated lung levels of lactate dehydrogenase (LDH), indicating occurrence of cell necrosis. We thus hypothesized that reduced lung IFN-β could contribute to necrotic cell death in a model of asthma exacerbations. Wild-type and IFN-β^−/−^ mice were given saline or house dust mite (HDM) intranasally for 3 weeks to induce inflammation. Double-stranded RNA (dsRNA) was then given for additional 3 days to induce exacerbation. HDM induced an eosinophilic inflammation, which was not associated with increased expression of cleaved caspase-3, cleaved PARP or elevated bronchoalveolar lavage fluid (BALF) LDH levels in wild-type. However, exacerbation evoked by HDM + dsRNA challenges increased BALF levels of LDH, apoptotic markers and the necroptotic markers receptor-interacting protein (RIP)-3 and phosphorylation of mixed linage kinase domain-like protein (pMLKL), compared to HDM + saline. Absence of IFN-β at exacerbation further increased BALF LDH and protein expression of pMLKL compared to wild-type. We demonstrate that cell death markers are increased at viral stimulus-induced exacerbation in mouse lungs, and that absence of IFN-β augments markers of necroptotic cell death at exacerbation. Our data thus suggest a novel role of deficient IFN-β production at viral-induced exacerbation.

## Introduction

Up to 80 percent of all asthma exacerbations are triggered by respiratory viral infections, which cause severe lower respiratory tract illness in asthmatics^1^. Pattern recognition receptors (PRRs) play a major role in innate immune responses to allergens and viruses^2,3^ and may also recognize components of dying cells^4^. Rhinoviruses produce double-stranded RNA (dsRNA) during replication, which is recognized by PRRs notably Toll-like receptor (TLR)-3 and retinoic acid-inducible gene I (RIG-I)-like receptors^5^. The result of activation of these PRRs involves the production and release of interferon (IFN)-β, which induces an antiviral state in surrounding cells^6^. It has been shown that primary cells from asthmatics may have a deficient ability to produce IFN-β at rhinoviral infection and dsRNA stimulation, the latter representing a given viral infection burden^7,8^. IFN-β is a multipurpose cytokine. In addition to its antiviral properties it can both induce cell death and, by contrast, promote cell survival in various cell types^9,10^. However, little is known regarding any association between IFN-β deficiency and occurrence of cell death in asthma or experimental models of asthma.

Virus infection-associated asthma exacerbations have been characterized by increased cell necrosis as reflected by released lactate dehydrogenase LDH^11^, a pan-cell-necrosis marker. A variety of cells in the asthmatic airways, including granulocytes and epithelial cells, may undergo necrosis at asthma exacerbations^12–14^. However, it is not known what modes of cell necrosis are involved. Eosinophil necrosis is clearly regulated in part by factors previously mistaken to specifically indicate apoptosis in these cells^15^. Apoptosis is a form of regulated cell death controlled by caspases and required for many physiological processes^16^. Apoptosis can be induced from extrinsic signals such as activators of cell surface death receptors or PRRs including TLR-3^17^. Once the initiator caspases get activated they cleave and activate caspase-3, which will execute apoptosis by proteolytic cleavage of several proteins including Poly (ADP-ribose) polymerase (PARP) involved in DNA repair^18^. If apoptotic cells are not phagocytosed they will undergo necrosis, which has been denominated as ‘secondary necrosis’. Necrosis is clearly induced by physical trauma such as heat damage or hypoxia. However, of special interest in disease is well-regulated necrosis^19^. Different modes of regulated necrosis have now been identified: secondary necrosis, necroptosis, and pyroptosis that all manifest with necrotic morphology^20^.

Necroptosis is a proposed form of programmed cell death that so far has not been clearly associated with human lung diseases although it is speculated to be involved in chronic obstructive pulmonary disease (COPD) and acute respiratory distress syndrome (ARDS)^21,22^. Necroptosis involves the proteins receptor-interacting protein (RIP)- 1, -3 and mixed linage kinase domain-like protein (MLKL). Upon activation, RIP1 and RIP3 form a complex called the necrosome, which phosphorylates MLKL to its active form that causes plasma membrane rupture. To avoid extensive necroptosis, the kinase activity of RIP1 and RIP3 is suppressed by full-length caspase-8^23^. Necroptosis has also been associated with inflammasome activation and subsequently interleukin (IL)-1β secretion and maturation^24,25^. Occurrence of necroptosis markers in asthma and animal models of asthma now awaits exploration.

We have recently developed a mouse model of viral stimulus-induced exacerbation of asthma with similarities to human exacerbations including increased bronchoalveolar lavage fluid (BALF) levels of LDH compared to allergic lung inflammation without exacerbation^26^. In this study, we test our hypotheses (A) that necroptosis occurs at viral induced exacerbations and (B) that IFN-β deficiency may be involved in increased lung necrosis. Part of the present results of these studies have previously reported in the form of abstracts^27^.

## Results

### Allergic airway inflammation induced by HDM does not involve LDH release or caspase-3 activation

**Figure 1:**
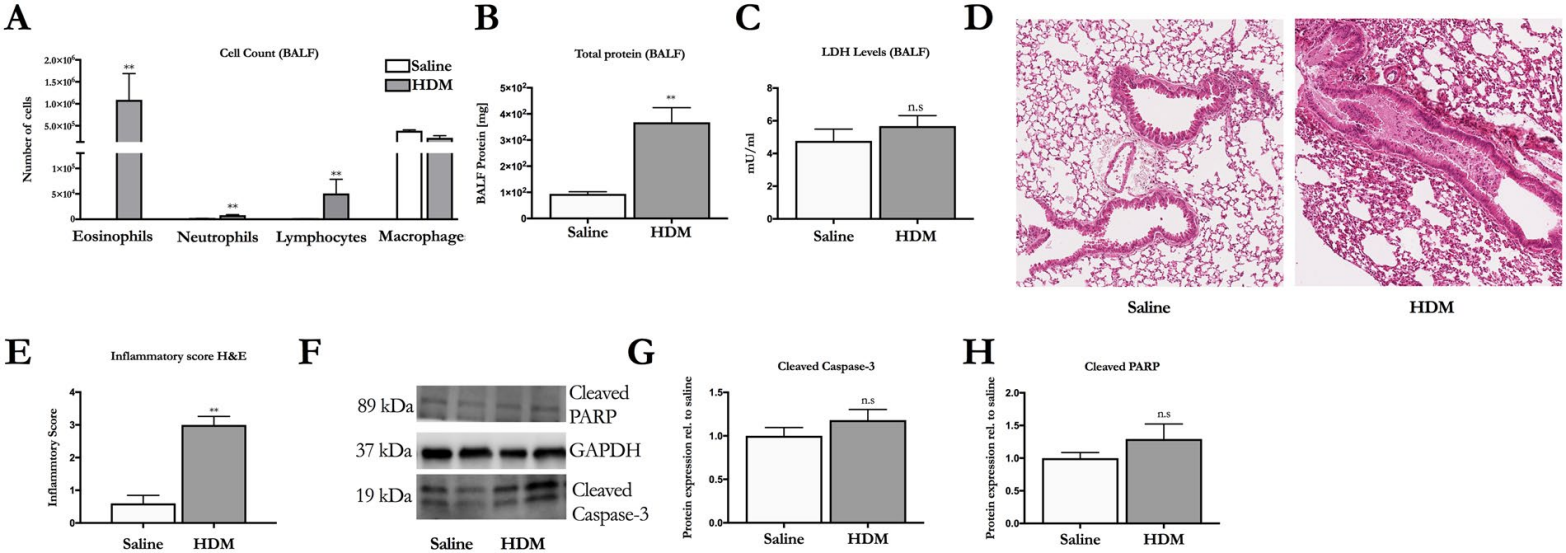
HDM induces lung inflammation. (**A**) Differential cell count in BALF (**B**) Total protein (**C**) Release of the pan-necrotic marker LDH in BALF (**D**) H&E staining of mice lung sections (**E**) Inflammation scored in lung tissue. (**F**) Representative immunoblots of cleaved caspase-3 and cleaved PARP. (**G**) Quantification of protein expression of cleaved caspase-3 and (**H**) cleaved PARP from immunoblots. Optical density was measured and bands related to housekeeping protein GAPDH and normalized towards saline. The data are presented as mean ± SEM (n = 5–6). *p < 0.05 vs saline, **p < 0.01 vs saline.

Mice where challenged with HDM or saline for three weeks to establish experimental asthma (Figure S1). HDM challenges induced an increase in total number of eosinophils, neutrophils and lymphocytes (Fig. 1A). There was also higher total protein levels in BALF in HDM challenged mice compared to saline challenged mice (Fig. 1B), which are in line with previously published data^26^. We could not detect any difference in the release of the cell death marker LDH in BALF after HDM challenges (Fig. 1C). Tissue staining with H&E of mouse lungs showed that HDM challenges increased perivascular and peribronchial infiltration of immune cells, and induced mucus production, which was not found in mice challenged with saline (Fig. 1D,E). We then analyzed protein expression of the apoptotic markers cleaved caspase-3 and cleaved PARP. There was no difference in protein expression of cleaved caspase-3 and cleaved PARP in mice challenged with HDM compared to saline (Fig. 1F–H).

### Increased expression of both apoptotic and necroptotic markers during viral stimulus-induced asthma exacerbation

**Figure 2:**
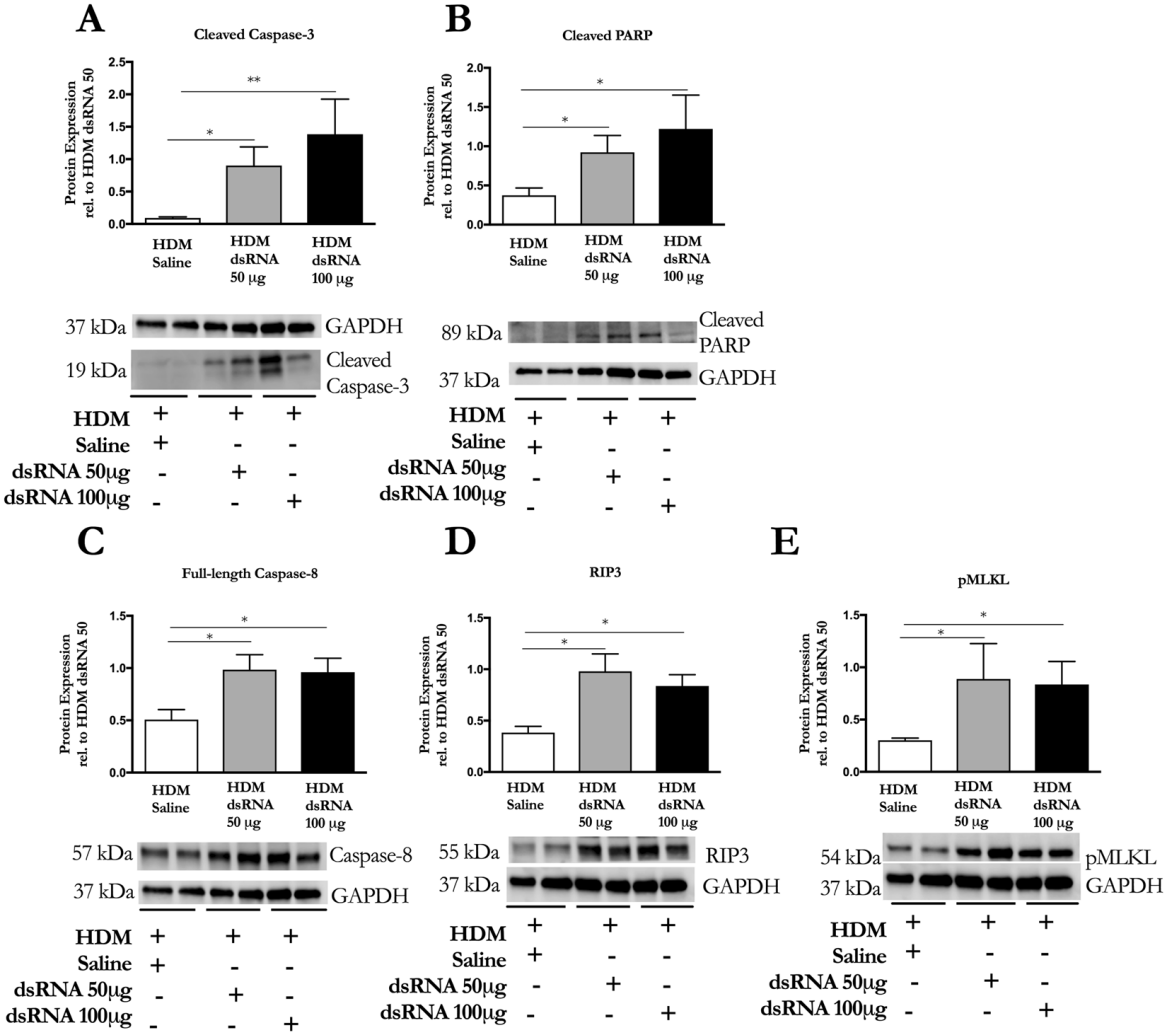
Exacerbating mice have increased expression of apoptotic and necroptotic markers. Representative immunoblots and quantification of (**A**) cleaved caspase-3, (**B**) cleaved PARP (**C**) full-length caspase-8 (**D**) RIP3 (**E**) pMLKL from homogenized lungs. Optical density was measured and bands were related to housekeeping protein GAPDH and normalized towards HDM:dsRNA 50. The data are presented as mean ± SEM (n = 5–6). *p < 0.05, **p < 0.01.

Asthma exacerbations have been associated with increased cell death, but the mechanism leading to cell death is largely unknown. Since we found that allergic airway inflammation induced by HDM developed without pronounced involvement of apoptosis or necrosis we wanted to examine the occurrence of various molecular cell death markers during viral-induced asthma exacerbation. We used a previously established mouse model of experimental asthma exacerbation where two different doses of dsRNA as a viral mimic (50 μg, 100 μg) or saline control were given intranasally to mice with established HDM-induced airway inflammation^26^ (Figure S1). We found that exacerbation evoked by both doses of dsRNA increased the apoptotic markers cleaved caspase-3 and cleaved PARP compared to HDM:saline challenged mice (Fig. 2A,B). Further, we found that both 50 μg and 100 μg dsRNA increased the expression of full-length caspase-8 (Fig. 2C). The expression of the necroptotic effector proteins RIP3 and phosphorylated MLKL were also increased to a similar level with both doses of dsRNA (Fig. 2D,E), indicating occurrence of necroptosis at exacerbation.

### Interferon-β deficiency increases BALF LDH levels at dsRNA-induced asthma exacerbation in mice

**Figure 3:**
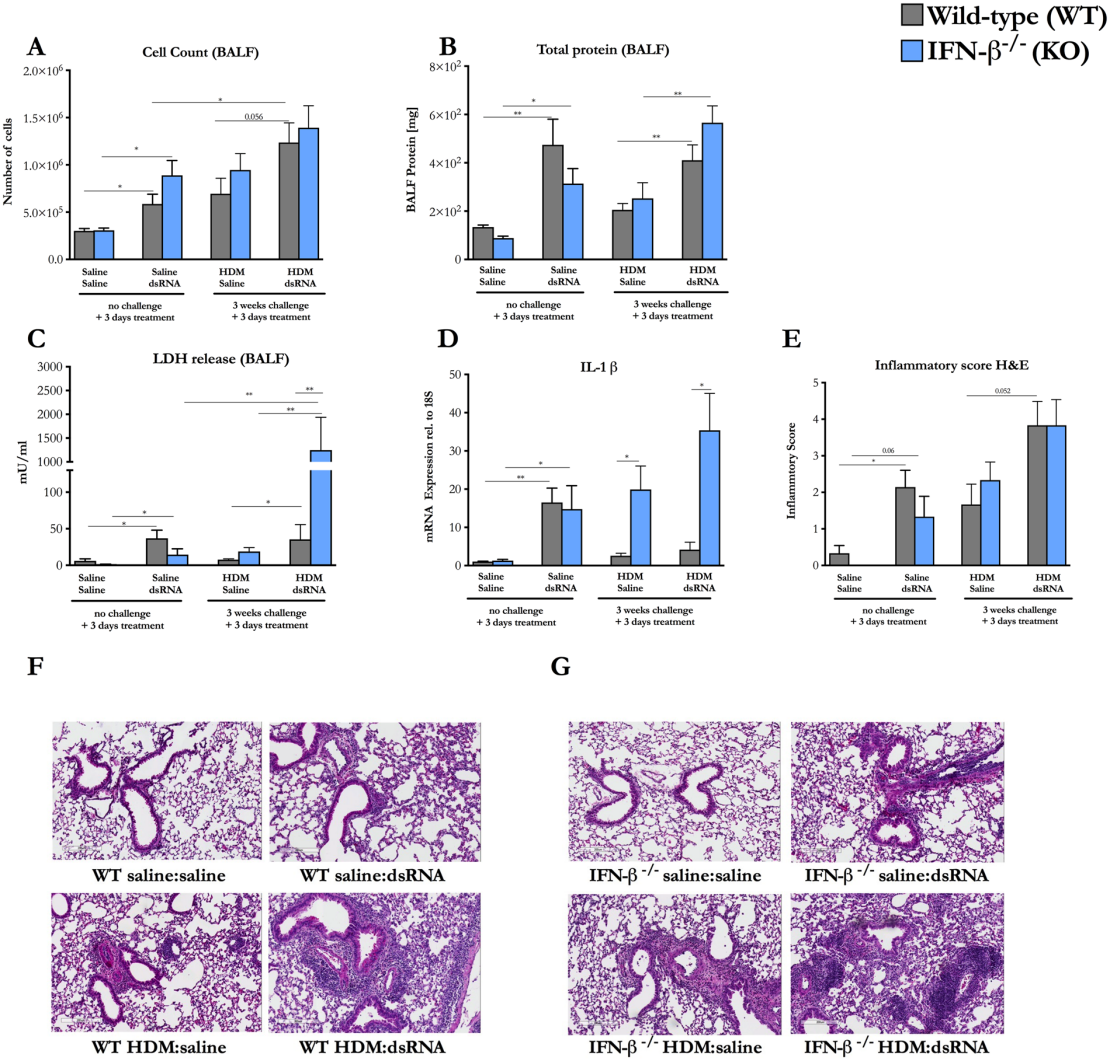
Interferon-β is protective against cell BALF LDH release at exacerbation. (**A**) Total cell count in BALF (**B**) Total protein in BALF (**C**) Release of the pan-necrotic marker LDH in BALF (**D**) Gene expression of IL-1β from lung homogenate. (**E**) Inflammation scored in lung tissue. (**F**,**G**) H&E staining of mice lung sections. The data are presented as mean ± SEM (n = 4–10) (n = 4–5 in control groups). *p < 0.05, **p < 0.01.

Having examined the effects of dsRNA on mouse lungs previously challenged with HDM for three weeks as regards to cell death markers we next performed a study with 50 μg dsRNA that included mice deficient in IFN-β. There was a trend towards increased total cell count in BALF in wild-type mice at exacerbation compared to HDM:saline challenged wild-type mice (Fig. 3A). There was also a higher total cell count in BALF at exacerbation compared to saline:dsRNA challenged wild-type mice, indicating that the combination of HDM and dsRNA produced an aggravated immune response (Fig. 3A). Total cell count in IFN-β^−/−^ mice had similar pattern as wild-type mice (Fig. 3A). In wild type mice at exacerbation there was a higher percentage of neutrophils compared to HDM:saline challenged wild-type mice, while the percentage of eosinophils was similar between the two groups (Figure S2B,D). However, saline:dsRNA challenged IFN-β^−/−^ had higher percentage of neutrophils, lymphocytes and eosinophils compared to wild-type mice with the same treatment (Figure S2B–D). Furthermore, there was a shift towards an increase of the percentage of lymphocytes, in the IFN-β^−/−^ mice at exacerbation compared to wild-type mice at exacerbation (Figure S2C). Total protein and LDH levels in BALF in wild-type mice were increased at exacerbation compared HDM:saline challenged wild-type mice (Fig. 3B,C). Similarly there were increased total protein and LDH release in BALF in IFN-β^−/−^ mice at exacerbation compared to IFN-β^−/−^ mice that where challenged with HDM:saline (Fig. 3B,C). Strikingly, there were much higher levels of LDH in BALF at exacerbation in IFN-β^−/−^ mice compared to wild-type mice, indicating occurrence of cell necrosis (Fig. 3C). This was accompanied by increased gene expression of IL-1β at exacerbation in IFN-β^−/−^ mice compared to wild-type mice at exacerbation (Fig. 3D). H&E staining showed a trend towards increased perivascular recruitment of immune cells in wild-type mice close to large airways at exacerbation compared to both HDM:saline and saline:dsRNA challenged wild-type mice (Fig. 3E,F). Tissue staining revealed comparable inflammation pattern in IFN-β^−/−^ mice (Fig. 3E,G).

### Lack of interferon-β increases pMLKL in HDM-challenged mice compared to wild-type mice

**Figure 4:**
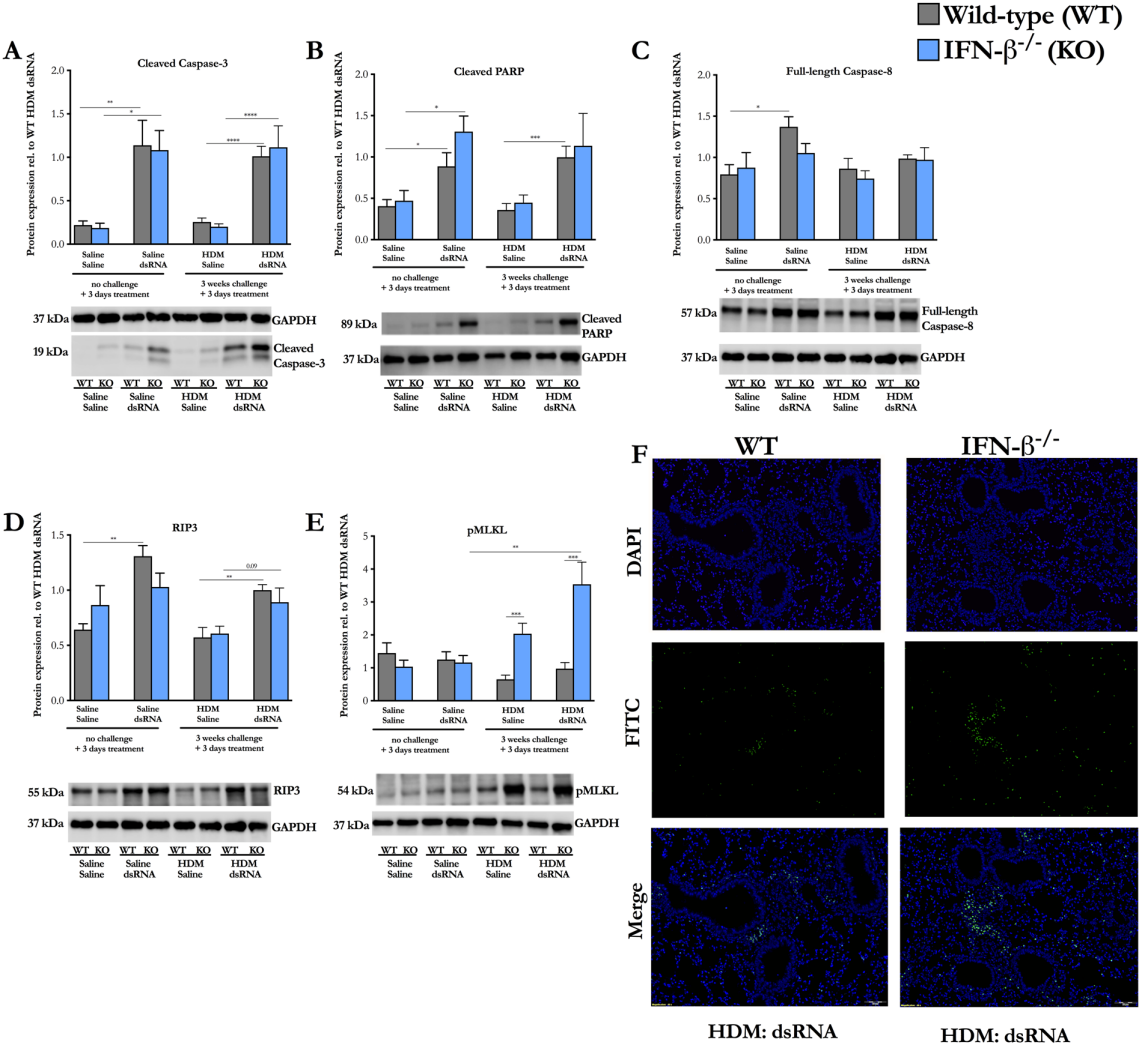
Interferon-β is protective against necroptosis. Representative immunoblots and quantification of (**A**) cleaved caspase-3 (**B**) cleaved PARP (**C**) full-length caspase-8 (**D**) RIP3 (**E**) pMLKL from homogenized lungs. Optical density was determined and bands were related to housekeeping protein GAPDH and normalized towards HDM:dsRNA. (**F**) Representative TUNEL-staining. Nucleus is stained blue. TUNEL-postive cells are stained green. The data are presented as mean ± SEM (n = 5–10). *p < 0.05, **p < 0.01, ***p < 0.001, ****p < 0.0001.

We then studied specific cell death markers in wild-type and IFN-β^−/−^ mice. We found that protein expression of the apoptotic markers cleaved caspase-3 and cleaved PARP were increased to similar extent in both saline:dsRNA and HDM:dsRNA challenged wild-type mice compared to wild-type mice not receiving dsRNA (Fig. 4A,B). In IFN-β^−/−^ mice, there was also a higher protein expression of cleaved caspase-3 and cleaved PARP in both saline:dsRNA and HDM:dsRNA challenged mice compared to IFN-β^−/−^ mice not receiving dsRNA (Fig. 4A,B), although cleaved PARP did not reach statistical significance at exacerbation. The protein expression of RIP3 was also increased in wild-type mice at exacerbation compared to HDM:saline challenged mice, however this was not the case for full-length caspase-8. In IFN-β^−/−^ mice, RIP3 expression also tended to be increased at exacerbation compared to HDM:saline challenged IFN-β^−/−^ mice, although not significant (Fig. 4D). There was a 3-fold higher protein expression of pMLKL, which causes cell membrane rupture at exacerbation in IFN-β^−/−^ compared to wild-type mice at exacerbation (Fig. 4E). TUNEL-positive cells were found in similar patterns in both wild-type and IFN-β^−/−^ mice at exacerbation (Fig. 4F).

## Discussion

This study addressed the occurrence of different modes of cell death and their dependence on IFN-β in a model of viral stimulus-induced asthma exacerbations. We demonstrate that the exacerbation is associated with increased markers of both apoptosis and necroptosis along with increased release of the pan-necrosis indicator, LDH. Furthermore, cell death indices at exacerbation were further increased in IFN-β deficient mice; the most conspicuous observation being a marked increase in LDH release together with an index of necroptosis and increased gene expression of IL-1β. These data are novel and of interest with regard to potential pathogenic roles of cell death and IFN-β deficiency in asthma, respectively.

We used HDM as inducer of a baseline allergic inflammation because this allergen is commonly involved in asthma. HDM induced inflammation interacts with the viral stimulus dsRNA to produce robust and reproducible exacerbation features including increased necrosis as reflected by increased BALF levels of LDH^26^. By the present regimen, HDM produced an eosinophilic inflammation with no signs of cell death. These results suggest that allergic lung inflammation is not always associated with aggravated cell death response. This observation suited the present focus on exacerbation. However, it is acknowledged that authors employing other HDM regimens and cell culture studies have reported that HDM has capacity to evoke cell death^28,29^ including markers of epithelial cell apoptosis that were not increased in this study. The present viral stimulus, dsRNA, is employed as a rhinovirus infection intermediate known to mimic biological effects of actual infection^30,31^. This study also reproduced central features of viral exacerbations reported previously and that agree with observations in human asthma including LDH release and mixed granulocyte and protein exudation features^11^. There is a further potential advantage with dsRNA challenges in exploratory studies comparing different interventions because it provides the opportunity of exposing the animals to a given pathogen burden. This goal may be difficult to achieve with actual infection, in particular when variations between animals in the antiviral response and pathogen resistance against virus occurs.

We demonstrated that two different doses (50ug and 100ug) of dsRNA challenges, administered to animals with an established HDM-induced allergic condition, produced significant increases in lung indices of apoptosis, cleaved caspase-3 and cleaved PARP, as well as necroptosis markers, RIP3, and pMLKL. These observations demonstrated involvement of regulated cell death in viral-induced exacerbations. The lower dose level of dsRNA (50ug) was therefore chosen for further studies of effects of dsRNA alone and for comparisons between wild-type mice and IFN-β deficient animals regarding exacerbation features.

In test systems involving cancer cells and macrophages, dsRNA has known effects on cell death indices reportedly involving both apoptosis and necroptosis^32,33^. Similarly to dsRNA, influenza infection has also been shown to induce both apoptosis and necroptosis^34^. Hence, it is not surprising that dsRNA increased LDH levels and markers of both apoptosis and necroptosis in wild-type mice in this study. Mice deficient in IFN-β showed amplified levels of LDH and the necroptotic marker pMLKL at exacerbation compared to their wild type counterparts. Furthermore, there was also higher levels of LDH and the necroptotic marker pMLKL in IFN-β^−/−^ mice at exacerbation compared to saline:dsRNA challenged IFN-β^−/−^ mice. In contrast, apoptotic markers were not altered in IFN-β^−/−^ mice at exacerbation. However, this finding may not exclude that secondary necrosis contributed to the high levels of LDH. A majority of apoptotic eosinophils in a model of severe asthma remained non-phagocytosed and underwent secondary necrosis, which along with direct cytolysis of eosinophils was associated with increased airway epithelial derangement and inflammation^35^. Our results suggest that virus in combination with allergy could lead to a more detrimental cell death response in those asthmatics with reduced IFN expression.

Currently, the cellular source of the released LDH during asthma exacerbations and experimental models of asthma is not known. Wild-type and IFN-β^−/−^ mice at exacerbation had similar pattern of TUNEL-positive cells. The TUNEL assay detects cells with fragmented DNA, and detects both apoptotic and secondary necrotic cells^36^. The TUNEL-positive cells were found in inflammatory foci, suggesting that they could be recruited inflammatory cells. Indeed, non-injurious resolution of inflammation in mucosal lined hollow organs such as the lungs is not dependent on apoptosis of inflammatory immune cells because the disease-driving cells in the airway wall are evidently eliminated through transmigration into the airway lumen for final clearance by mucociliary transport^37^. Shedding of epithelial cells is a hallmark of asthma likely contributing to pathogenesis of the disease. Especially large numbers of epithelial cells are shed at exacerbations. They appear in sputum and BALF as conglomerates called Creola bodies consisting of 10 cells or more including alive and dead cells^13^. However, it is of note that epithelial cells do not have to die before being shed but be released through down-regulation of adhesion proteins in intercellular junctions^38^.

Necroptosis may have contributed to the high LDH levels because pMLKL was increased at exacerbation in IFN-β deficient animals. Yet, RIP3 did not follow same pattern as pMLKL, which remains to be explained. pMLKL-induced necroptosis has been associated with inflammasome activation and increased IL-1β expression^24^ Interestingly, also in this study we demonstrated increased IL-1β expression along with the increased pMLKL, potentially extending the role of necroptosis to involve promotion of IL-1β dependent features of asthma exacerbation^39^.

Previous studies have focused on the importance of IFN-β as an antiviral agent and its deficiency at viral-induced asthma exacerbations^40^. Based on the present findings we suggest a novel additional role of IFN-β deficiency as a regulator of necrosis and necroptosis at exacerbation of asthma. How IFN-β may affect cell death in context of asthma has previously been limited to observations *in vitro*, where IFN-β was required for an apoptotic fate of viral-infected cells^7^. It has been reported that IFN-β knockout mice had higher levels of cytokines potentially promoting necrotic cell death, including TNF-α in central nervous system compared to wild-type mice^41^. Our results showed that the inflammation in wild-type mice and IFN-β^−/−^ mice was not much different at exacerbation at least at one specific time-point. Interestingly, we found that IFN-β deficient mice challenged with three weeks of HDM had increased expression of pMLKL compared to wild-type. In contrast, the apoptotic markers were mainly induced by dsRNA in both wild-type and IFN-β^−/−^ mice. Hence, the inflammation induced by HDM in mice that are deficient in IFN-β may have a dysfunctional tissue repair mechanism involving necroptosis, which might lead to a prolonged inflammation. The present finding thus provides a basis for future exploration of time course aspects of pathogenic factors emanating from necroptosis at asthma exacerbations. Future studies are also warranted to validate the present novel findings with dsRNA in experimental exacerbations involving HDM and live rhinovirus infections.

Occurrence of necroptosis in lung diseases has only recently started to be explored^42^. This is the first paper to our knowledge that shows involvement of necroptosis and hence potential pathogenic cell death in asthma models. In COPD patients, elevated expression levels of RIP3 has been observed in lung epithelial cells compared to controls but pMLKL, considered the most appropriate marker of necroptosis, was not studied^20,43^. Diseases that have been suggested to involve necroptosis have also increased incidence of other forms of necrosis. This may be expected because the cell death pathways, as reflected by currently employed molecular markers, are highly intertwined^44^. How much necroptosis and necrosis contribute individually to driving inflammation needs further studies. Similarly the present discovery of a novel role of IFN-β as regulator of necrosis/necroptosis at viral induced exacerbations needs validation in future studies.

## Materials and Methods

Additional information about materials and methods is provided in the supplementary material.

### Animals

A mixture of female and male C57BL/6 wild-type mice and IFN-β^−/−^ mice were maintained in an animal facility at Lund University. Animal experiments were approved by the Malmö/Lund Animal Experimental Ethics Committee at the Lund District Court in Sweden (approval number M36-13). All animal care and protocols were governed by the European Parlement and Council Directive 2010/63/EU, the Swedish Animal Welfare Act (Djurskyddslag 1988:534), the Swedish Animal Welfare Ordinance (Djurskyddsförordning 1988:539) and Institutional Animal Care and Use Committee (IACUC) guidelines. The IFN-β^−/−^ mice have previously been evaluated during infection with Sendai virus and in a experimental model of autoimmune encephalomyelitis^41,45^. The mice were fed *ad libitum*. Experimental asthma and asthma exacerbation in mice were induced as previously described^26^. Shortly, the mice were challenged with 25 μg/mouse dose HDM (Greer, Lenoir, USA) or saline intranasally 3 times/week for 3 weeks in order to establish experimental asthma. For the exacerbation model, HDM or saline challenged mice received 50 μg or 100 μg dsRNA {polyinosine-polycytidylic acid [Poly(I:C)]; (InVivogen, San Diego, USA)} or saline intranasally as control for 3 additional days. Mice were divided in seven groups; saline, HDM, saline/saline, saline/dsRNA, HDM/saline, HDM/dsRNA 50 μg and HDM/dsRNA 100 μg. The experiment was terminated three days after the last saline/HDM challenge for the two first groups, and 24 hours after the last saline/dsRNA or saline administrations for the other groups (Figure S1).

### Bronchoalveolar lavage fluid

BALF was obtained by rinsing the lungs with PBS. BALF was centrifuged and the supernatants were then used to measure LDH release. The cell pellet from BALF was then resuspended in PBS and analyzed for total cell count with NucleoCounter (Chemometec, Allerod, Denmark). 50 000 cells where loaded to a cytospin funnel and where centrifuged at 450 g for 6 minutes. The cytospin slides where than stained with May-Grünwald Geimsa and analyzed under microscope for differential cell count.

### Lung dissection and preparation

The left lung lobes were fixed in 4% formaldehyde (Histolab, Gothenburg, Sweden) and paraffin embedded followed by sectioning. The sectioned lungs where then used for terminal deoxynucleotidyl-mediated dUTP nick end labeling (TUNEL) and hematoxylin and eosin (H&E) staining. The H&E slides where analyzed under light microscope and a score (1–6) was given reflecting the degree of lung inflammation. Sections with no obvious cell infiltrate were scored 0. All slides were analyzed blindly. The right lobe was snap frozen in liquid nitrogen until usage for western blot analysis or RT-qPCR. The right lung lobes were weighed and homogenized mechanically by using an OmniPrep Rotor Stator Generator (Omni International, Waterbury, USA). For western blot the lungs where additionally chemically lysed with lysis buffer (1% TritonX-100, 10 mM Tris-HCl, 50 mM NaCl, 5 mM EDTA, 30 mM sodium pyrophosphate, 50 mM NaF, 0.1 mM Na_3_VO_4_) together with 1% protease and 1% phosphatase inhibitor cocktail (Sigma-Aldrich, Stockholm, Sweden). Total protein was measured with Pierce BCA assay (Thermo Scientific, Waltham USA).

### Statistical analysis

The data are presented as mean±SEM. The statistical difference between-group comparisons were made using the Mann–Whitney U test. P-values of <0.05 were considered statistically significant. Statistics were performed by GraphPad Prism version 6.0 g software (GraphPad Software).

### Data availability

The authors declare that all the data supporting the findings of this study are available from the corresponding author on request.

## Electronic supplementary material

Supplementary manuscript
