# Supplementary material for: Interferon-β deficiency at asthma exacerbation promotes MLKL mediated necroptosis
Source: Sci Rep. 2018 Mar 9;8:4248. doi: 10.1038/s41598-018-22557-6 (PMC5844912; doi:10.1038/s41598-018-22557-6)
Supplement: Supplementary file 1 — Supplementary manuscript [file 41598_2018_22557_MOESM1_ESM.pdf]

Supplementary

Interferon-β deficiency at asthma exacerbation promotes MLKL mediated necroptosis

Authors: Samuel C. Cerps# [1], Mandy Menzel# [1], Irma Mahmutovic Persson[1], Leif Bjermer[2], Hamid Akbarshahi [1,2], Lena Uller* [1]

1: Unit of Respiratory Immunopharmacology, Department of Experimental Medicine, Lund University
2: Respiratory Medicine and Allergology, Lund University.

* **Corresponding author:**

Lena Uller,
lena.uller@med.lu.se,
phone: 0046 222 04 13
Sölvegatan 19,
BMC D12, Lund

Materials and Methods

Western-blot analysis

Equal protein amount of lung homogenate were diluted in 1:1 volume sample Laemmli buffer (Sigma-Aldrich, Stockholm, Sweden) and boiled for 5 min. The extracts were loaded on a 15 μL well Mini-protean TGX gel (Bio-Rad, Stockholm, Sweden) and separated by gel electrophoresis using a mini protean tetra cell system electrophoresis chamber (Bio-Rad, Stockholm, Sweden). The proteins were then transferred to TransBlot Turbo mini nitrocellulose membrane (Bio-Rad, Stockholm, Sweden) with Trans-blot Turbo transfer system (Bio-Rad, Stockholm, Sweden) and then blocked in 5% milk or 5 % BSA (Sigma-Aldrich, Stockholm, Sweden), TBS-T (20 mM Tris-base, 137 mM NaCl, 0,1 % Tween 20). Following primary antibodies were used and diluted 1/1000 in TBS-T with 5% BSA (Sigma-Aldrich, Stockholm, Sweden): pMLKL (abcam#196436), cleaved caspase-3 (Cell Signaling, Beverly, USA, #9664), cleaved PARP (Cell Signaling, Beverly, USA #9544), caspase-8 (Cell Signaling, Beverly, USA #8592), RIP3 (Cell Signaling, Beverly, USA #15828), and GAPDH (Cell Signaling, Beverly, USA #5174). Membranes were incubated with primary antibodies at 4 C°. Membranes where then washed in TBS-T with 5% milk or 5% BSA, for 2x 15 minutes and then washed in TBS-T for additional 15 minutes. Anti-rabbit secondary antibodies #31460 (Thermo Scientific, Waltham, USA) or anti-rabbit # 7074 (Cell Signaling, Beverly, USA) conjugated to HRP were directed towards primary antibodies and incubated for 1 hour. pMLKL were stripped for RIP3 and caspase-8 with western blot stripping buffer (Thermo Scientific, Waltham, USA). Proteins were visualized with West Femto SuperSignal detection kit (Thermo Scientific, Waltham, USA) and optical density from the bands was detected by using LI-COR odyssey Fc imager system (LI-COR, Lincoln, USA). Ratio of optical band density between samples and GAPDH were calculated in Microsoft Excel and normalized towards saline or wild-type HDM/dsRNA. The mean of the normalized optical density are presented.

TUNEL staining

In situ cell death detection kit fluorescein (Roche, Mannheim, Germany) was used for TUNEL analysis. Sections were deparaffinized and gradually rehydrated. Antigen-retrieval was performed by addition of 20 μL of 20 mg/ml proteinase K (Sigma-Aldrich, Stockholm, Sweden) during 20 minutes at 37 °C. TUNEL mixture was then added to all sections and incubated 1 hour at 37 °C.

Sections were then washed and Nucblue (DAPI) (Thermo Scientific, Waltham, USA) was added to sections for 5 min in order to stain the nucleus. Tissue sections were finally mounted using aqua pertex (HistoLab, Gothenburg, Sweden) and analyzed under fluorescent microscope (Nikon Eclipse 80i, Tokyo, Japan)

LDH assay

LDH levels in BALF were measured by cytotoxicity detection kit according to manufactures protocol (Roche, Stockholm, Sweden).

RNA isolation and gene expression quantification by RT-qPCR

RNA extraction kit Nucleospin® RNA II (Macherey-Nagel, Düren, Germany) was used to isolate total RNA from right lung lobe homogenate and reverse transcribed to cDNA with Precision Nanoscript Reverse Transcription kit (PrimerDesign, Southampton, UK). RT- qPCR was run on an Mx3005P qPCR system (Stratagen, La Jolla, USA). Primers were obtained from (Qiagen, Stockholm, Sweden) and (PrimerDesign, Southampton UK). Analysis of samples was performed by the ΔΔCt method and related to 18S expression as previously described (1). Groups where normalized to saline/saline group.

References

(1) Livak, K. J., & Schmittgen, T. D. (2001). Analysis of relative gene expression data using real-time quantitative PCR and the 2– ΔΔCT method. *methods*, *25*(4), 402-408.

Figure S1

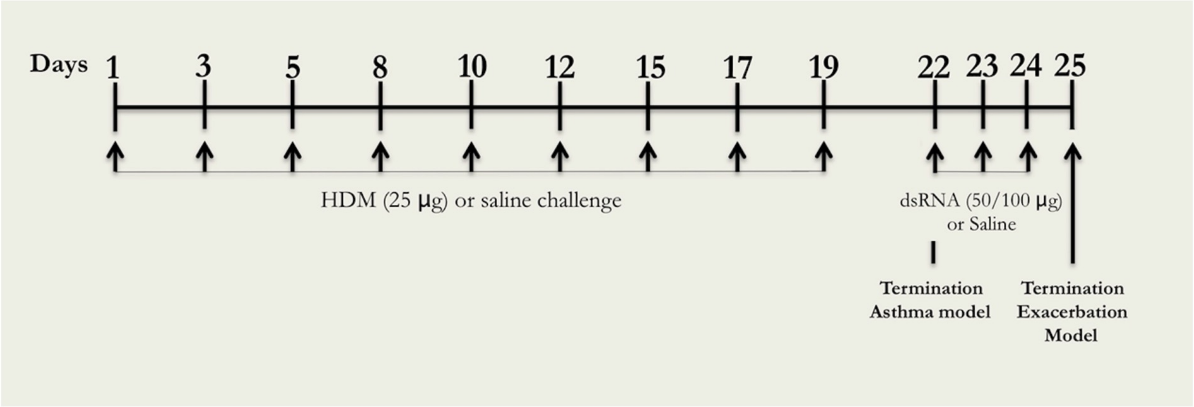
Days
1
3
5
8
10
12
15
17
19
22
23
24
25
HDM (25 μg) or saline challenge
dsRNA (50/100 μg) or Saline
Termination Asthma model
Termination Exacerbation Model

Figure S2

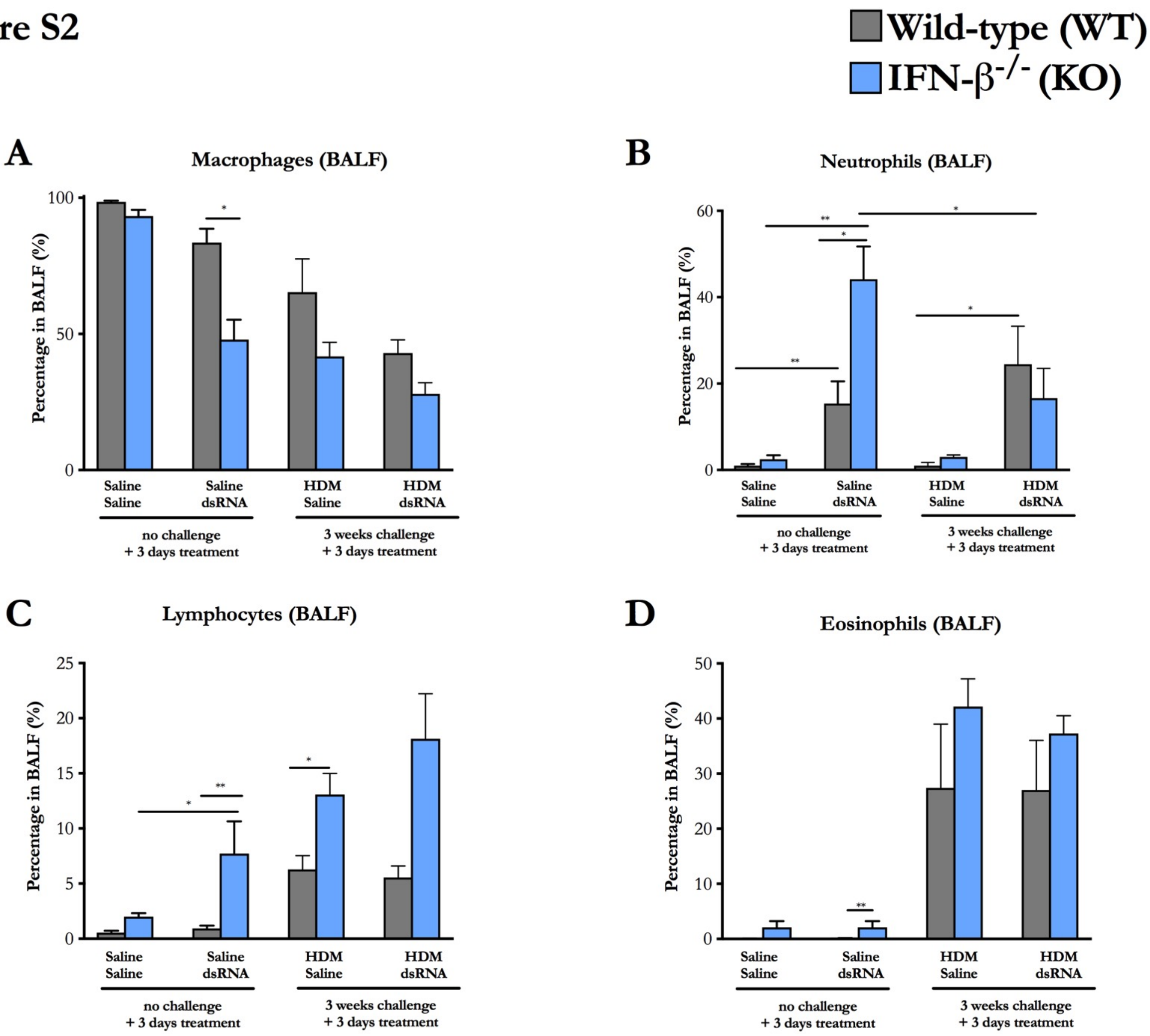

Wild-type (WT)
IFN-β-/- (KO)
A
Macrophages (BALF)
Percentage in BALF (%)
100
50
0
Saline Saline
Saline dsRNA
HDM Saline
HDM dsRNA
no challenge + 3 days treatment
3 weeks challenge + 3 days treatment
B
Neutrophils (BALF)
Percentage in BALF (%)
60
40
20
0
Saline Saline
Saline dsRNA
HDM Saline
HDM dsRNA
no challenge + 3 days treatment
3 weeks challenge + 3 days treatment
C
Lymphocytes (BALF)
Percentage in BALF (%)
25
20
15
10
5
0
Saline Saline
Saline dsRNA
HDM Saline
HDM dsRNA
no challenge + 3 days treatment
3 weeks challenge + 3 days treatment
D
Eosinophils (BALF)
Percentage in BALF (%)
50
40
30
20
10
0
Saline Saline
Saline dsRNA
HDM Saline
HDM dsRNA
no challenge + 3 days treatment
3 weeks challenge + 3 days treatment

Supplementary figure legends

Figure S1: **Study Design.**

Mice were challenged with 25 μg HDM or saline intranasally 3 times/week for 3 weeks. For the exacerbation model, HDM or saline challenged mice received 50 μg or 100 μg dsRNA or saline for 3 additional days.

Figure S2: **Differential cell count in BALF.**

(**A**) Percentage macrophages in BALF. (**B**) Percentage neutrophils in BALF. (**C**). Percentage lymphocytes in BALF. (**D**) Percentage eosinophils in BALF. The data are presented as mean+SEM (%) (n=4-10).
